# Acknowledgment to Reviewers of *Journal of Functional Morphology and Kinesiology* in 2020

**DOI:** 10.3390/jfmk6010010

**Published:** 2021-01-22

**Authors:** Journal of Functional Morphology Kinesiology Editorial Office

**Affiliations:** MDPI AG, St. Alban-Anlage 66, 4052 Basel, Switzerland

Peer review is the driving force of journal development, and reviewers are gatekeepers who ensure that *JFMK* maintains its standards for the high quality of its published papers. Thanks to the cooperation of our reviewers, in 2020, the median time to first decision was 23 days and the median time to publication was 42 days. The editors would like to express their sincere gratitude to the following reviewers for their precious time and dedication, regardless of whether the papers were finally published:
Abalasei, Aurelia BeatriceLavender, AndrewAberer, FelixLee, HaneulAdams, KentLee, Sang WookAdom, TheodosiaLind, ErikAguiar, Andreo FernandoLjubisavljevic, MilosAkbar, UmerLongo, StefanoAlfieri, AndreinaLoreto, CarlaAlmeida Marinho, DanielLouis, JulienArsoniadis, Gavriil G.Lucariello, AngelaAssanelli, DeodatoLucienne, VonkAyán, CarlosLuis Hernandez-Davo, JoseAziz, Abdul RashidLukaszuk, JudithBadau, AdelaLystad, Reidar P.Bailey, ChrisMacaluso, FilippoBalzeau, AntoineMaciejczyk, MarcinBanfi, GiuseppeMainwaring, Lynda M.Barrea, LuigiMaly, TomasBenson, PhilipManning, Gerald S.Beretta-Piccoli, MatteoManuel, Rodríguez-HuguetBest, RussellMartín Valero, RocíoBeyer, KyleMartin, EricBogataj, ŠpelaMartinho, JoséBogdanis, GregoryMartins, JoãoBonilla, Diego A.Mason, LauraBrito, João PauloMaugeri, GraziaBudde, HenningMcErlain-Naylor, StuartCalcagno, GiuseppeMcKenna, Malachi J.Camacho-Cardeñosa, AlbaMcMahon, John J.Campa, FrancescoMcNeill, ConorCampbell, Kody R.Mengarelli, AlessandroCandow, DarrenMeulstee, Jene W.Cangiano, BiagioMichel, Marina EvrardCarlino, ElisaMielgo-Ayuso, JuanCarrera Quintanar, LucreciaMinervini, GiuseppeCarriot, JérômeMoccia, MarcelloCasa, DouglasMolgó, JordiCasagrande Figueiredo, VandréMolina-Hidalgo, CristinaCè, EmilianoMora-González, JoséChambers, MaryMoral-García, José EnriqueChan, Kuei-HuiMoretti, AntimoChang, Shin-TsuMurillo, SerafínChang, Shuo-HsiuNagarajan, PrabakaranChen, Yung-ShengNavalta, JamesChin, Kok YongNegra, YassineChmielewska, DariaNosrat, SanazChtourou, HamdiOliva, FrancescoChulvi-Medrano, IvánOrlacchio, AntonioCicchella, AntonioPadulo, JohnnyCoco, MarinellaPalma, AntonioCollado, Pilar SánchezPaoli, AntonioConceicao, FilipeParraça, José Alberto Frade MartinsCondello, GiancarloPastor, JesúsCortis, CristinaPastora-Bernal, Jose ManuelCoulson, SamanthaPatwardhan, Avinash R.Cumming, SeanPereira, AnaD. Blasio, AndreaPiacentini, Maria FrancescaD’Amico, Agata GraziaPiancino, MariaDe La Cruz Sánchez, ErnestoPirttiniemi, PerttiDe Sire, AlessandroPitino, DarioDi Cagno, AlessandraPrieske, OlafDi Giunta, AngeloPrimack, WilliamDi Paolo, Carlo D.Proia, PatriziaDickin, ClarkPrue, GillianDomínguez, RaulPueo, BasilioDrum, ScottPugliese, GabriellaDrury, BenjaminRachon, DominikDuquette, PierreRama, LuísEbraheim, NabilRambold, Holger A.Ehrenpreis, Eli DRauch, Jacob T.Fekete, GusztávRavalli, SilviaFeria Madueño, AdriánReglödi, DóraFerlazzo, NadiaRinaldi, SimonFernandes, John F. T.Rocha Vaz, JoãoFernando De Rezende, LuizRojas Valverde, DanielFerrara, ZacharyRossi, AlessioFerraz, Ricardo Manuel PiresRoy, Brian D.Fisher, JamesSams, MattFormanowicz, DorotaSato, KimitakeFoster, CarlSeibert, Franz JosefFranchi, Martino V.Sekulic, DamirFrancois, MoniqueSeo, Dae YunFry, AndySeo, YongsukFulford, JonathanSeok, HyunFusco, AndreaSerra, LídiaFusco, AugustoShalfawi, Shaher A. I.Gallardo, LeonorSharp, Matthew H.García-Ceberino, Juan M.Sidiropoulos, KonstantinosGarcía-Romero, JeronimoSilver, TobinGentil, PauloSinghal, KunalGeorgian, BadicuSirico, FeliceGlynn, Nancy WSong, PingGobbo, StefanoSpringer, FabianGollie, Jared M.Stanforth, Philip RGoodworth, AdamStilli, DonatellaGörgülü, RecepŠtrumbelj, BoroGreco, GianpieroSyczewska, MalgorzataGrisold, WolfgangSyed-Abdul, Majid MufaqamGuerra, GermanoSzabo-Reed, Amanda N.Guglielmino, ClaudiaTerzis, GerasimosHatchett, AndrewTessitore, AntonioHaubruck, PatrickTorres-Sánchez, IreneHelms, Eric R.Tsai, Kuen-JerHernandez-Esteban, PabloTurkmen, MutluHernández-García, RaquelUgolini, AlessandroHough, JohnVadalà, GianlucaHsieh, Ming-FaVamvakis, AnastasiosHunt, MelissaVan De Tillar, RolandHussain, GhulamVaz, Marco AurélioHyde, ChristianVescovi, Jason D.Jaime, SalvadorWadhi, TanujJiannine, LiaWieringa, FokkoJin, DiWirth, KlausJosé Tauler Riera, PedroWunsch, KathrinJoshi, Pushpa RajYamakado, KotaroKabasakalis, AthanasiosYasar, ZerbuKavanagh, JustinYasue, AkihiroKeller-Ross, Manda L.Zaffagnini, StefanoKellis, EleftheriosZahálka, FrantišekKendall, KristinaZając, AdamKirschneck, ChristianZancanaro, CarloKoppelaar, HenkZanuso, SilvanoKrueger, EddyZare, MohsenKuan, GarryZhan, YiqiangLamb, KevinZiegenfuss, TimLamberti, NicolaZieliński, RafałLane, AndrewZotti, FrancescaLarussa, TizianaZubac, DamirLatsios, George


